# A Review of Finite Element Analysis and Artificial Neural Networks as Failure Pressure Prediction Tools for Corroded Pipelines

**DOI:** 10.3390/ma14206135

**Published:** 2021-10-15

**Authors:** Suria Devi Vijaya Kumar, Michael Lo Yin Kai, Thibankumar Arumugam, Saravanan Karuppanan

**Affiliations:** Mechanical Engineering Department, Universiti Teknologi PETRONAS, Seri Iskandar 32610, Malaysia; michael_19000348@utp.edu.my (M.L.Y.K.); thibankumar@gmail.com (T.A.); saravanan.karuppanan@utp.edu.my (S.K.)

**Keywords:** artificial neural network, finite element analysis, failure pressure prediction

## Abstract

This paper discusses the capabilities of artificial neural networks (ANNs) when integrated with the finite element method (FEM) and utilized as prediction tools to predict the failure pressure of corroded pipelines. The use of conventional residual strength assessment methods has proven to produce predictions that are conservative, and this, in turn, costs companies by leading to premature maintenance and replacement. ANNs and FEM have proven to be strong failure pressure prediction tools, and they are being utilized to replace the time-consuming methods and conventional codes. FEM is widely used to evaluate the structural integrity of corroded pipelines, and the integration of ANNs into this process greatly reduces the time taken to obtain accurate results.

## 1. Introduction

The oil and gas industry encompasses multiple highly complex services and facilities that involve exploration, production, and refinement of petroleum products. These services often have multiple facilities that span large distances. Transportation of hydrocarbons, often in fluid form, relies heavily on pipelines due to the large distances. Pipelines are preferred as they are the most cost-efficient and safe mode of transport for oil and natural gas [1,2]. It is crucial that a pipeline is always capable of withstanding the operating pressures of the transport system. Otherwise, major problems that result in the disruption of operations may arise, especially when necessary precautions are not taken [3]. The integrity of a pipeline is compromised when pipeline degradation occurs on the walls of the pipeline. One of the leading causes of pipeline degradation is corrosion. Corrosion defects lead to the premature failure of pipelines, which is the failure of a pipeline at pressures lower than the initial operating pressure.

Among various types of corrosion, uniform corrosion, pitting corrosion, and erosion corrosion are some of the most common types that occur in pipelines [4]. Uniform corrosion is identified as an even corrosive attack over the pipeline wall. On the other hand, pitting corrosion occurs in a localized area, and it has been proven to be more destructive [1,5]. In the presence of fluid flow, pitting corrosion may lead to erosion corrosion, which causes the defect to increase in size due to the turbulence. A corrosion defect is classified as pitting corrosion if its length and width are less than or equal to 3 times the uncorroded wall thickness [3,6,7,8].

Based on the DNV-RP-F101 (DNV) assessment guideline for corroded pipelines, corrosion defects can be categorized into three categories: are single defect, interacting defect, and complex-shaped defect. A single defect is a defect that is sufficiently isolated from neighboring defects such that there is no interaction between them. Its failure pressure is independent of the other defects that are present in the pipeline. Interacting defects are defined as two or more defects aligned in the longitudinal or circumferential direction that interact with one another. The resulting failure pressure is lower than that of the individual single defects. A complex-shaped defect results from the combination of different colonies of interacting defects or a single defect [9]. Since it is challenging to completely prevent the occurrence of corrosion, it is critical to monitor the condition of the pipeline. Continuous assessment of the residual strength of the pipeline is necessary to ensure that the transport system is being operated at safe levels of pressure so that the corrosion defects do not lead to catastrophic failures.

Conventional residual strength assessment methods generally result in conservative pipeline failure pressure prediction due to the assumptions and safety factors. This results in unnecessary pipeline maintenance and repairs. However, with the use of computer-aided failure analysis methods such as the finite element method (FEM), the accuracy of pipeline failure pressure prediction could be enhanced [10]. However, carrying out finite element analysis (FEA) can be computationally expensive. To overcome this, an artificial neural network (ANN) can be utilized. Hence, this paper reviews the capabilities of ANNs being integrated into FEM as tools for fast yet accurate corroded pipeline failure pressure prediction.

## 2. Conventional Residual Strength Assessment Methods

Over the years, various methods have been developed to assess the failure pressure of corrosion-affected pipelines. This effort was driven by the need for an accurate failure pressure prediction method in the industry. Fitness-for-purpose analysis of pipelines used in the oil and gas industry requires detailed technical assessment of a defect to ensure that the structure can serve its purpose as long as the failure conditions are not reached [6]. In the industry, several methods are widely used to predict the failure pressure of corroded pipelines. Some of the commonly employed methods are summarized in Table 1. In these models, the corrosion defect parameters that are considered are the corrosion depth and longitudinal length. The equations in these methods are independent of the width of the corrosion.

The ASME B31G method is based on the NG-18 equation and is one of the methods that is commonly used in the industry. This method assumes the defect idealization based on the length of the corrosion. Short corrosion defects where L ≤ $\sqrt{20Dt}$ are assumed to consist of corrosion regions that are of a parabolic shape with a curved bottom. As for long corrosion defects where L > $\sqrt{20Dt}$, it is assumed that the corroded region is rectangular in shape with a flat bottom [11].

By redefining the Folias factor and flow stress equations of the ASME B31G method, the modified ASME B31G method was developed. In this method, an arbitrary shape correction factor is applied instead of the parabolic area assumption. The factor 2/3 was replaced with 0.85 in the failure pressure prediction equation as presented in Table 2. This enables the method to be applied to corrosion defects that are longer than the limits given in the ASME B31G method. The SHELL 92 method also utilizes the same Folias factor as the ASME B31G method. However, this method produces predictions that are relatively conservative due to the flow stress assumption of the method [12].

Besides, the RSTRENG method, also known as the effective area method, is used in assessing defects up to 0.8 *t*. This method represents the corrosion defect region with a river bottom profile that enables a failure pressure prediction with greater accuracy using the discrete method [11,12]. These methods are based on the fundamental NG-18 equation and the calculation of the flow stress and Folias factor differs based on the assumptions for each method. The respective Folias factor and flow stress equations are summarized in Table 3. On the contrary, the pipe corrosion criterion (PCORRC) method is based on a finite element study that was validated using burst test results, and the corroded pipe strength (CPS) method is based on the weighted depth difference method [13].

**Table 1:** Common pipe failure pressure assessment methods [6,9,13].

Method	Fundamental Equation	Governing Assumption	Material Restriction	Defect Idealization
ASME B31G	NG-18	Flow stress-dependent mechanism causes the pipe failure. Therefore, it can be described by the tensile properties of the pipe.	Low toughness	Parabolic or rectangular
Modified B31G	NG-18		Low toughness	Mixed shape
SHELL 92	NG-18		-	Rectangular
RSTRENG	NG-18			Effective area
DNV RP-F101	NG-18	Plastic collapse (plastic flow) controls pipe failure where the ultimate tensile strength is the flow stress.	Moderate toughness	Rectangular
Corroded Pipe Strength (CPS)	Extensive numerical studies (validated against test data)		Moderate toughness	Step shape
PCORRC criteria			Moderate to high toughness	Elliptical

**Table 2:** Common pipe failure pressure assessment method equations [8,9,10].

Method	Failure Pressure, *P_f_*
ASME B31G	$\{\begin{array}{ll} \sigma_{f}\frac{2t}{D}\left[\frac{1-\left(2/3\right)\left(d/t\right)}{1-\left(2/3\right)\left(d/t\right)/M}\right], & \mathrm{for}\;L\;\leq \;\sqrt{20Dt}\\ \sigma_{f}\frac{2t}{D}\left[1-\frac{d}{t}\right], & \mathrm{for}\;L>\sqrt{20Dt} \end{array}$
Modified B31G	$\sigma_{f}\frac{2t}{D}\left[\frac{1-0.85\left(d/t\right)}{1-0.85\left(d/t\right)/M}\right]$
RSTRENG	$\sigma_{f}\frac{2t}{D}\left[\frac{1-A_{d}/A_{0}}{1-A_{d}/A_{0}M}\right]$
SHELL 92	$\sigma_{f}\frac{2t}{D-t}\left[\frac{1-d/t}{1-d/tM}\right]$

However, the conventional corrosion defect assessment procedures result in predictions that are conservative due to the incorporation of safety factors in the calculations [14,15]. When compared with all other assessment methods, the DNV RP-F101 assessment method is found to be the most comprehensive method [16]. The failure pressure of a pipe with a single corrosion defect subjected to internal pressure only is calculated using Equation (1). To consider external stress, the correction factor, H_1_, is determined using Equation (2). Combining Equations (1) and (2), Equation (3) is formed, which allows for the failure pressure prediction of a pipe with a single corrosion defect subjected to internal pressure and external stress.

(1) $$P_{fs,DNV}=\gamma_{m}\left(\frac{2\;t\;\sigma_{UTS}}{D-t}\right)\left(\frac{1-\gamma_{d}\left[{\left(d/t\right)}_{measured}+\varepsilon_{d}StD\left(d/t\right)\right]}{1-\frac{\gamma_{d}\left[{\left(d/t\right)}_{measured}+\varepsilon_{d}StD\left(d/t\right)\right]}{\sqrt{1+0.31{\left(l/\sqrt{Dt}\right)}^{2}}}}\right)$$

(2) $$H_{1}=\frac{1+\left(\frac{\sigma_{L}}{\xi \;\sigma_{UTS}}\right)\left(\frac{1}{1-\left(d/t\right)\theta }\right)}{1-\left(\frac{\gamma_{m}}{2\;\xi \;\left[1-\left(d/t\right)\theta \right]}\right)\left(\frac{1-\gamma_{d}\left[{\left(d/t\right)}_{measured}+\varepsilon_{d}StD\left(d/t\right)\right]}{1-\frac{\gamma_{d}\left[{\left(d/t\right)}_{measured}+\varepsilon_{d}StD\left(d/t\right)\right]}{\sqrt{1+0.31{\left(l/\sqrt{Dt}\right)}^{2}}}}\right)}$$

(3) $$P_{fs,DNV}=\gamma_{m}\left(\frac{2\;t\;\sigma_{UTS}}{D-t}\right)\left(\frac{1-\gamma_{d}\left[{\left(d/t\right)}_{measured}+\varepsilon_{d}StD\left(d/t\right)\right]}{1-\frac{\gamma_{d}\left[{\left(d/t\right)}_{measured}+\varepsilon_{d}StD\left(d/t\right)\right]}{\sqrt{1+0.31{\left(l/\sqrt{Dt}\right)}^{2}}}}\right)H_{1}$$

Traditional approaches such as the Monte Carlo simulation (MCS) method with the help of a nondestructive examination system are used to predict the probability of failure of the pipelines. The MCS is based on the concept of numeric sampling assisting in developing probabilistic models [17]. In essence, the MCS generates a great number of cases and criteria value conversions for each case [18]. Besides its flexibility and unlimited analyses, it enables the modeling of interdependent relationships between input and output variables [19,20]. However, this method takes up a great amount of time to produce results. In the presence of numerous variables bounded by various constraints, a lot of time is required for the computation of the solution [21,22,23,24,25]. Besides, this method results in solutions that are not exact but depend on the number of repeated runs [20].

In recent years, machine learning and finite element method (FEM) have been researched to replace the time-consuming methods and conventional codes. The failure pressure predictions obtained by utilizing these tools are more accurate and less conservative compared to the conventional approaches [26]. In this effort, artificial neural networks (ANNs) are being widely used together with FEM as corroded pipe failure pressure prediction methods [1,27,28]. In comparison to the MCS method, which can take more than three hours to produce results, an ANN can produce accurate results in a matter of seconds [1,27].

## 3. Artificial Neural Network as a Corroded Pipeline Failure Pressure Prediction Tool

Gurney [29] defined an ANN as an interconnected assembly of simple processing elements called nodes, and the processing ability of the network is stored in the interunit connection strengths called weights which are obtained by learning a set of training patterns. Highly complex nonlinear systems that produce accurate results can be efficiently modeled [30]. Various learning algorithms can be utilized in machine learning depending on the nature of the training data and the expected output results, as summarized in Table 4 [31]. ANNs are becoming increasingly popular as prediction tools to predict the failure pressure of corroded pipelines due to their ability to recognize and infer from patterns without requiring explicit instruction [32,33]. However, to predict accurate outcomes, an ANN has to be trained sufficiently using reliable training data [34].

**Figure 1:**
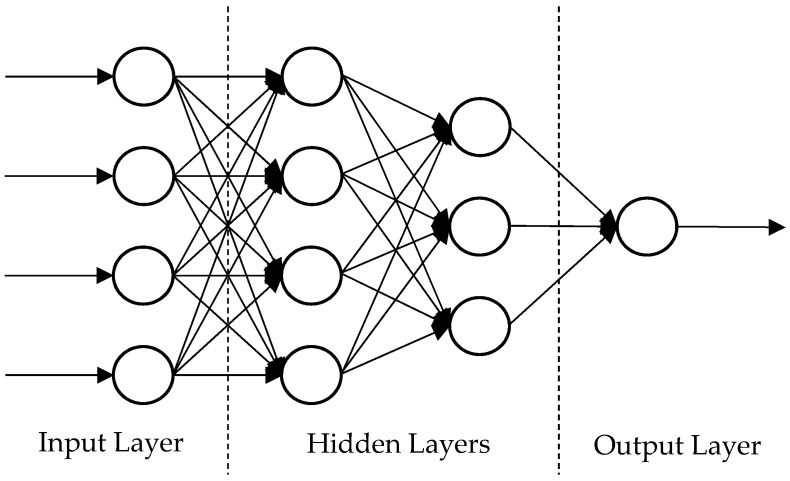
A traditional feedforward neural network (FFNN) model with two hidden layers.

The architecture of an ANN also depends on the type of data and desired output. Some of the commonly used ANN architectures are summarized in Table 5. Among them, FFNN is mostly applied in predicting the failure pressure of corroded pipelines. This type of ANN architecture is modeled to learn from paired datasets where the model learns from one or more inputs and the corresponding output of the training dataset. An FFNN is straightforward and suitable to be used for producing one output. The architecture of an FFNN contains an input layer, hidden layer, and output layer, as illustrated in Figure 1. Generally, an FFNN is used with the Levenberg–Marquardt back-propagation algorithm to train the model as it not only performs efficiently but also requires less time and fewer epochs for convergence [40].

Various researchers have applied this method to develop prediction models that are able to predict pipeline failure and replace time-consuming methods such as the MCS. Wen et al. [27] utilized an ANN with a back-propagation algorithm to develop a methodology for the reliability assessment of corroded natural gas pipelines. Using a case study from Shuai et al. [28], they compared the results obtained using their ANN model and MCS. It was proven that the utilization of an ANN greatly reduces the time taken to obtain results without compromising on the accuracy of the prediction [41,42].

Every ANN uses activation functions that determine the output of a neural network. Generally, they can be classified into two categories, namely linear and nonlinear activation functions. Some of the commonly used activation functions are summarized in Table 6. The sigmoid or logistic function and rectified linear unit are usually used as the activation function for the prediction of pipeline failure pressure due to corrosion as they cater for outputs with positive values only.

Some researchers have utilized ANNs to develop a model for the failure prediction of pipelines by taking into account the physical, mechanical, operational, and environmental factors. This approach has shown promising results proving the robustness of ANN models when it comes to predicting the residual life of pipelines. Zangenehmadar et al. [51] used this approach in their research to determine the useful life of pipelines using the Levenberg-Marquart back-propagation algorithm. Their ANN model was able to predict the useful life of a pipeline with an error percentage of less than 5%. However, Shirzad et al. [52] emphasized in their paper that when such factors are considered, an ANN model cannot be easily generalized due to the lack of real-life data. Based on El-Abbasy et al. [22] in such models, a comprehensive input is needed to ensure that the model is accurate. Hence, large datasets of real-life cases need to be gathered and used as training datasets.

When following this approach to predict the failure pressure of pipelines, the issue of having a limited amount of real-life data can be overcome using the finite element method (FEM) to generate training data for the ANN model. In a study conducted by Xu et al. [10], the authors utilized FEA to obtain the failure pressure of a pipeline with interacting defects. Their study proved that FEA can be used to predict the failure pressure of pipelines with a relative error percentage of less than 1% when compared to actual burst test results. Hence, FEA can be used to generate as many reliable ANN training data as needed depending on the availability of time and facilities.

## 4. Finite Element Method (FEM) as a Corrosion Defect Assessment Method

FEM is one of the numerical methods for solving differential equations of engineering problems. The method was originally developed to solve structural mechanics problems, and it has since been extended to other branches of engineering and science, such as heat transfer, fluid dynamics, and electromagnetism. FEM is the numerical method of choice to evaluate the integrity of a pipeline as this method can be employed in widely available commercial FEM software such as ANSYS, ABAQUS, and ALTAIR HyperWorks [1,2,27,53,54].

FEM is a Level 3 evaluation method for corroded pipelines according to the American Society of Mechanical Engineers [55]. There are different levels of evaluation for corroded pipelines, and hence the levels of evaluation are chosen based on the type and number of data available. Level 0 evaluation method is carried out using reference tables of calculated failure pressure from equations developed by established standards and codes from Level 1 evaluation. Level 1 evaluation method requires the pipe parameters and defect geometries to be measured and calculated in accordance with standards and codes such as ASME B31G, Modified ASME B31G, and DNV to evaluate the failure pressure. Level 2 evaluation method or effective area method uses a more detailed measurement of corrosion defect to predict the failure pressure, commonly through standards such as “API 579 Level 2” assessment and software package such as RSTRENG. Level 3 evaluation uses numerical methods such as FEM to predict accurate failure pressure. Level 3 evaluation method considers greater detail of information in comparison to the previous levels.

**Figure 2:**
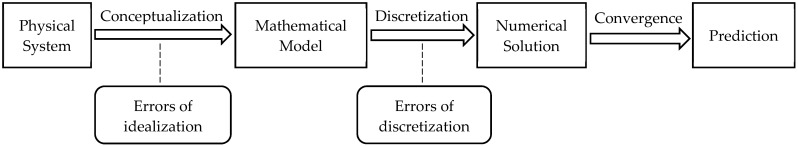
Development of FEM [56].

In FEM, physical systems of interest in physical reality are conceptualized into mathematical models to predict responses of interest. To assess corrosion defects, FEM idealizes the pipe body and geometries of corrosion defect with internal and external load exerted on the pipe to be represented in mathematical models such as nodes. The nodes are inputted with data of physical properties, such as modulus of elasticity, and boundary conditions, such as loads and constraints. These inputs are then computed through selected numerical solutions suited for the type of FEM analysis [56]. It is known that FEA can provide accurate solutions depending on the error associated with its mathematical models, known as the error of idealization, and the error associated with a numerical solution, known as the error of discretization. The development flow of FEM is illustrated in Figure 2.

Errors of idealization or modeling error can be reduced when input data for the finite element mathematical model represent the real conditions of the corroded pipeline accurately. As such, accurate assessment of corroded pipe through FEM will require information such as type of analysis, material properties, defect geometry, loadings, boundary conditions, and failure criterion to enable a comprehensive and accurate prediction of failure pressure using FEM.

The strength of corroded pipelines is commonly assessed through structural analysis, specifically nonlinear structural analysis as opposed to linear structural analysis. In structural mechanics, the relationship between input and output is linear for a linear system as opposed to a nonlinear system [57]. To determine the failure pressure of corroded steel pipelines accurately, the nonlinear material property of steel needs to be considered. The nonlinear stress–strain curve is inputted into the elements when meshing the finite element model [3,58]. A material is considered to have failed when its stress reaches its ultimate tensile strength (σ_UTS_).

There are two types of elements commonly used to model a steel pipeline in FEM, namely shell elements and solid elements. When analyzing hollowed cylindrical structures, shell elements reduce the dimension of the problem to 2D, whereas solid elements are employed in 3D analyses. In 2013, Sadowski and Rotter explored the viability of the elements in analyzing the failure of cylindrical tubes under global bending and concluded that solid elements are more suitable for tubes as thick as r/t = 10 (ratio of radius of curvature, r, to thickness, t) while shell elements are more economical when r/t ≥ 25 in terms of computational time without noticeable loss in accuracy [59]. Shell elements are commonly used to generate models of thin pipes and plates. When modeling thick subsea pipelines, solid elements would allow for more accurate meshing in comparison to shell elements.

Aging pipelines are often plagued by corrosion with irregular shapes and complex geometry. The complex geometries of corrosion defects are often idealized into simple shapes in FEM for ease of assessment. The most common idealized defect shapes are rectangular, elliptical, parabolic, and in some cases conical shapes [14,60,61,62,63,64]. Wang et al. [65] investigated the failure pressure difference between finite element models with a rectangular defect and a spherical defect. The study found that there is an insignificant difference in failure pressure prediction between the two idealized defects. Amaya-Gómez et al. [13] stated that conventional standards commonly propose rectangular defect shapes to assess pipeline for an additional level of conservatism; however, elliptical defect shapes are preferred in studies using FEM to assess corroded pipelines. Mokhtari and Melchers’ [66] work proved that failure pressure prediction in FEM using an idealized semielliptical defect produced a lower average error and coefficient of variance than an idealized rectangular defect, which is supported by experimental and numerical investigation done by Netto et al. [64].

Internal corrosion is more prevalent than external corrosion in pipelines that transport hydrocarbons in pipeline corrosion failure incidents [67]. Despite the statistical difference, both internal corrosion and external corrosion ultimately reduce the thickness of the pipe wall. The behaviors of internal and external corrosion defects are similar, as observed in full-scale burst tests and FEA [68]. Therefore, the location of modeled defect (interior or exterior) has little effect on the results of FEA.

FEM requires a material failure criterion to be defined to properly predict the failure pressure of a corroded pipeline [69,70,71]. The ultimate tensile stress (UTS)-based von Mises failure criterion is commonly adopted in the FEM framework of current research using commercial finite element software such as ANSYS and ABAQUS. A common failure criterion for plastic collapse of a corroded pipeline defines failure when the von Mises stress reaches UTS throughout the ligament (wall thickness) of the corrosion defect [72]. However, it was found that the UTS-based failure criterion overestimates the failure pressure of high-toughness pipelines [73]. This could be attributed to UTS-based approaches’ lack of consideration for the strain hardening exponent of a steel pipeline [70].

The error of discretization is largely dependent on the model’s element discretization. There are different types of elements of various geometrical shapes for different dimensional problems and basic functions such as displacement, stress, and strain, which are necessary to compute the data of interest within acceptable error bounds. Thus, appropriate meshing at areas of interest, such as at corrosion defect regions and pipe segments, is needed to balance between evaluation accuracy and computational time consumption.

FEA predictions of failure pressure are more accurate when compared to existing standards and codes [62,64,74]. Conventional assessment standards such as ASME B31G, Modified ASME B31G, DNV, RSTRENG, and PCORRC are found to be conservative with their failure pressure estimation as these analytical and empirical models are based on simplifications and assumptions [74,75]. FEM is not only accurate, but the method can manipulate geometrical data of corrosion defects and introduce complex loads onto the pipe model with ease, allowing improved evaluation of failure pressure in addition to faster development of assessment methods for corroded pipelines in comparison to experimental full-scale burst tests.

## 5. Integration of Finite Element Method and Artificial Neural Network as Residual Strength Prediction Tool

FEM is more accurate for the assessment of failure pressure in a corroded pipeline when compared to the conventional assessment standards [1,2,3,27,53]. However, FEM is time-consuming to carry out, and a comprehensive parametric study is computationally intensive [1,27]. Carrying out extensive parametric studies using FEM is not practical, and this is where machine learning has proven to be useful [29]. ANNs can be used to overcome this issue by following three approaches identified in this study. There are a few approaches to how the integration between these two tools can be achieved, as summarized in Table 7.

The first approach is by incorporating the ANN directly into the framework of the FEM. ANN and FEM are powerful prediction tools that have proven to produce highly accurate results while consuming less calculation or computation time compared to just FEA [1,27,76]. Researchers have taken advantage of these tools and explored the possibilities of integrating both tools to produce better and more efficient prediction models in various fields. Javadi and Tan [77] integrated ANN in their FEA to predict the relationship between the stress and strain of a material. Their resulting predictions proved the adaptability and efficiency of the integrated tools. They concluded that an ANN is capable of substituting complex mathematical models in FEM. Their research is supported by Hashash et al. [78], who addressed numerical implementation problems pertaining to the incorporation of ANN directly into the FEM framework. Their study proved that the approach results in good convergence characteristics and robustness of the tools.

In addition, the direct incorporation of ANN into the FEM process was further researched by Gulikers in 2018. He developed a framework that allows substructure homogenization of complex material properties through a constitutive model captured by ANN [76]. Data generated through a series of FEM simulations of a chosen substructure were used to train the ANN. The neural network predicts the mechanical behavior of the substructure as a function of the parameters it was trained with. The trained ANN was then integrated into FEM as a user material subroutine, where the homogenized substructure is represented as an element. The computational time of FEM with integrated ANN is instantaneous, where any loading combinations were evaluated in about 3 s. The FEM with integrated ANN constitutive model was accurate in its estimation as the maximum observed verification error was below 5%. His work provided the groundwork for the possibility of an ANN integrated framework in the application of FEA to estimate the failure of steel pipelines.

The second approach is by using FEM to generate training data for the development of an ANN model. As mentioned in various studies, an ANN requires sufficient training to ensure the accuracy of the model. Often in reality, many data are inaccessible, and it costs a great deal to run experiments. In such cases, parametric studies can be carried out using FEM to generate a sufficient number of data that is required for the performance of the ANN model [79,80,81,82]. The resulting ANN can be used to produce results by directly receiving a set of inputs that represent the real-life scenario.

The third approach is by developing an empirical equation that represents the developed ANN based on its weights and biases. This way there is no need for advanced software to be utilized. Tohidi and Sharifi in 2016 utilized this approach and furthered their research by developing an empirical solution to predict the residual ultimate strength of steel based on the ANN model that was trained. The equation that was formulated proved to be a simple yet accurate assessment method [83].

**Table 7:** Types of approaches for the integration of FEA and ANN.

Author	Field	Summary	Methodology
Javadi and Tan (2003) [77]	Computer Science	ANN is incorporated in FEM to substitute conventional constitutive material model.	ANN as part of the FEA framework.
Hashash et al., (2004) [78]	Civil Engineering	Models constituting ANN are incorporated in the FEM to address issues related to its numerical implementation.	
Gulikers (2018) [76]	Aerospace	Data generated through a series of FEA of a chosen substructure were used to train the ANN. The trained ANN was then integrated into the FEM as user material subroutine.	
Low and Chao (1992) [79]	Electrical Engineering	ANN models for solving problems related to inverse electromagnetic fields are developed using FEM to generate training data.	The ANN is developed based on training data generated using FEA.
Gudur and Dixit (2008) [80]	Mechanical Engineering	ANN to produce optimum parameters for process modeling is developed using FEM to generate training data.	
Umbrello et al., (2008) [81]	MechanicalEngineering	An ANN was developed to predict residual stresses and optimal conditions during steel processing using data generated using FEM for training and validation of the model.	
Shahani et al., (2008) [82]	Mechanical Engineering	An ANN model is developed to substitute time-consuming simulation process using data generated from FEM to train the model.	
Tohidi and Sharifi (2016) [83]	Civil Engineering	An empirical model is developed to predict the residual ultimate strength based on the ANN model.	An empirical solution is derived based on the ANN model trained using data generated from FEA.
Vijaya Kumar et al., (2021) [1]	Mechanical Engineering	An empirical model is developed to predict the failure pressure of an API 5L X80 pipe based on an ANN model.	
Lo et al., (2021) [27]	Mechanical Engineering	An empirical model is developed to predict the residual strength of an API 5L X65 pipe based on an ANN model.	

Similarly, the failure pressure of corroded pipelines can be estimated using this approach. Most research has been done on single corrosion defects; however, only a few studies on interacting corrosion defects have been conducted. The DNV code caters for single defects subjected to internal pressure and compressive stress and interacting defects subjected to internal pressure only. In reality, interacting defects are subjected to both internal pressure and compressive stress due to the harsh surrounding environments. Besides, DNV is recommended for medium-toughness pipes and may result in an inaccurate failure pressure prediction if used for high-toughness steel pipes [9]. This is where FEA can be utilized to provide reliable burst pressure predictions that can be used as training data for an ANN model. The finite element model can be validated against full-scale burst test results from past research and be used to generate new training data to be fed into the ANN model.

In 2007, Silva et al. utilized this approach to study the relationship between interacting corrosion defects and the pipe burst pressure using FEA and ANN where FEA was used to generate training data for the ANN. In their study, they concluded that the combination of both FEA and ANN to assess the structural integrity of corroded pipelines is a promising and efficient method [84]. In 2015, an assessment procedure was proposed for predicting the failure pressure of X80 pipelines with interacting corrosion defects by integrating FEA and ANN [85]. This approach was followed by Xu et al. in 2017 to study the effect of corrosion defect geometry on the failure pressure of a corroded pipe using the integration of FEA and ANN. In their research, they applied appropriate meshing and boundary conditions to their finite element model to ensure its accuracy. The resulting FEA predicted the failure pressures with a relative error of less than 1%, and their ANN model predicted the failure pressures of pipelines with interacting defects with a relative error of less than 2% [10]. However, in their research, they did not consider the compressive stresses acting on the pipe.

Lo et al. [27] and Vijaya Kumar et al. [1] furthered their research in this area by incorporating axial compressive stress acting on a pipe. In their study, the developed ANN was used to derive an empirical equation that was represented in matrix form. The equation was developed as a function of normalized axial compressive stress, normalized defect depth, length, and spacing. Both studies proved that the developed equations could predict the failure pressure of a corroded pipe accurately with an error percentage of less than 5% when compared to full-scale burst tests.

Based on the findings, it can be said that the integration of ANN in FEA greatly improves computing time compared to using FEA alone. A conventional FEA simulation takes up to 43,000 s, while it took only 3 s using the ANN incorporated FEA in the research of Gulikers in 2018 [76]. The generation of FEA results as training data for the development of ANN greatly increases the accuracy of the model as predictions obtained using FEA are less conservative compared to the conventional assessment methods [79,80,81,82]. Empirical solutions can then be derived from the weights and biases of the trained ANN model [1,27,83]. By doing so, a Level 3 corrosion assessment method can be reduced to a Level 1 complexity without compromising on the accuracy of the results.

In time-critical situations, ANN can provide results in a matter of seconds, unlike FEM which is time-consuming. Besides, by representing the ANN as an empirical equation, no advanced software or area of expertise is needed to carry out the assessment as the calculations can be carried out simply using just a spreadsheet [27].

## 6. Conclusions

The summary of the literature review and its findings are given as follows:

Conventional residual strength assessment methods are generally conservative in their predictions due to various simplifications and assumptions made during their development.

FEM is used extensively to evaluate the structural integrity of corroded pipelines due to its ability to accurately model corrosion defects and make predictions with high accuracy. However, FEM is computationally expensive and time-consuming to carry out comprehensively.

ANNs have been widely adopted in various fields for their predictive capabilities, development, and deployment speed. Their applications in assessing corroded pipelines are unlimited, especially in predicting the structural integrity of corroded pipelines.

An ANN can overcome the shortcomings of FEM through the integration of both methods.

The findings showed promising outcomes in terms of predicting the failure pressure of corroded pipelines in a short amount of time without compromising on the accuracy of the results by implementing an ANN as part of the assessment framework.

## Figures and Tables

**Table 3:** Flow stress and Folias factor determination for NG-18-based assessment methods [12,13].

Method	Flow Stress, *σ_f_*	Folias Factor, M
ASME B31G	1.1SMYS	$\sqrt{1+0.8\left(\frac{L^{2}}{Dt}\right)}$
Modified B31G	SMYS+68.95 MPa	$\{\begin{array}{ll} \sqrt{1+0.6275\left(\frac{L^{2}}{Dt}\right)-0.003375{\left(\frac{L^{2}}{Dt}\right)}^{2},} & \mathrm{for}\;L\;\leq \;\sqrt{50Dt}\\ 3.3+0.032\left(\frac{L^{2}}{Dt}\right), & \mathrm{for}\;L>\sqrt{50Dt} \end{array}$
RSTRENG	SMYS+68.95 MPa	$\sqrt{1+0.6275\left(\frac{L^{2}}{Dt}\right)-0.003375{\left(\frac{L^{2}}{Dt}\right)}^{2}}$
SHELL 92	$0.9\sigma_{UTS}$	$\sqrt{1+0.8\left(\frac{L^{2}}{Dt}\right)}$
DNV RP-F101	$\sigma_{UTS}$	$\sqrt{1+0.31\left(\frac{L^{2}}{Dt}\right)}$

**Table 4:** Machine learning paradigms [33,35,36,37,38,39].

Learning Paradigms	Algorithms	Remarks
Supervised learning	- Linear regression - Logistic regression - Linear discriminant analysis - K-nearest neighbors - Trees - Artificial neural network - Support Vector Machines - Back-propagation	- Expected output is presented to the network - All data points are used to train the network - Applicable to sequential data such as pattern or speech recognition
Unsupervised learning	- K-means clustering - Hierarchical clustering	- Expected output is not presented to the network - The system discovers and adapts to the structural features in the input pattern - Applicable to estimation problems and statistical distributions
Semisupervised learning	- Baum–Welsh hidden Markov Model - Graph-based kernels	- Combination of supervised and unsupervised learning - Expected output is not presented to the network - Only indicates if the output is correct or incorrect - Unlabeled dataset size should be substantially larger than the labeled data - Applicable in fields such as speech analysis and protein sequence classification
Reinforcement learning	- Propagating 1-nearest-neighbor - Markov decision process	- Involves interaction with the surrounding environment - Applicable in speech recognition and gaming (games that involve human interaction such as chess)

**Table 5:** Common ANN Architectures [30,31,32,33,34,35,36,37,38,39,40,41,42,43,44,45,46,47,48,49,50].

Architecture	Function
Feedforward neural network (FFNN)	Theoretically models the relationship between the input and output based on the training dataset [30].
Radial basis function (RBF)	Similar to an FFNN but uses radial basis activation function [31].
Recurrent neural networks (RNNs)	Uses data with no timeline and is a suitable option for advancing or completing information [32].
Long/short-term memory (LSTM)	Contains memory cell that overcomes the exploding gradient problem and learns complex sequences in the form of music or art [33].
Gated recurrent units (GRU)	Similar to LSTM but is faster and easier to run [34].
Autoencoders (AEs)	Used to encode data by compressing them [35].
Variational autoencoders (VAEs)	Relies on Bayesian mathematics pertaining to probabilistic inference to rule out improbable relations among inputs and outputs [36].
Denoising autoencoders (DAEs)	Used for noisy data where the model can be trained to learn details rather than the broader features of a data [37].
Sparse autoencoders (SAEs)	Used to extract details and small features from a given dataset [38].
Deep belief networks (DBNs)	Used to represent data as a probabilistic model, classify data, and generate new data [39].
Convolutional neural networks (CNNs)	Used for image or audio processing [40].
Deconvolutional networks (DNs)	Reversed convolutional networks, also called inverse graphics networks [41].
Deep convolutional inverse graphics networks (DCIGNs)	Used to model complex transformations on images [42].
Generative adversarial networks (GANs)	Two networks working together with one generating content and the other judging the contents [43].
Liquid state machines (LSMs)	Used to create a spiking-like pattern where there is a change in the output only when a certain threshold is reached [44,45].
Echo state networks (ESNs)	Similar to FFNN but utilizes random connections within the nodes [46].
Deep residual networks (DRNs)	Used in learning patterns that are up to 150 layers deep [47].
Capsule network (CapsNet)	Used in transferring information about an input using Hebbian learning, the values of which correct predictions of output in the next layer [48].
Kohonen networks (KNs)	Used to classify data without supervision by utilizing competitive learning [49].
Attention networks (ANs)	Used to visualize insight into which input features correspond with what output features [50].

**Table 6:** Activation functions used in ANN [35].

	Activation Function	Equation	Range
Linear	Linear function	f(x) = x	−infinity to infinity
Nonlinear	Sigmoid or logistic function	$a\left(x\right)=\frac{1}{1+e^{-x}}$	0 to 1
	Tanh or hyperbolic Tangent function	f(x) = tanh(x)	−1 to 1
	Rectified linear unit (ReLU)	f(x) = max(0, x)	0 to infinity

## Data Availability

Data sharing is not applicable for this article.
